# Comprehensive Experimental and DFT Studies on the Effect of Sodium and Calcium on Heavy Metals Adsorption Performance by Quartz During Coal Combustion

**DOI:** 10.3390/molecules30244792

**Published:** 2025-12-16

**Authors:** Jiangze Luo, Ziyang Zhang, Zhiwei Fan, Zhuozhi Wang, Xuegang Zhang, Xian Wei

**Affiliations:** 1 Sichuan Engineering Research Center for Titanium Alloy Advanced Manufacturing Technology, Panzhihua University, Panzhihua 617000, China; 2 School of Chemical Engineering and Technology, Hebei University of Technology, Tianjin 300401, China

**Keywords:** sodium, calcium, heavy metals, quartz, DFT, coal combustion

## Abstract

Quartz is capable of capturing heavy metals (HMs); however, alkaline metals compete with HMs for active adsorption sites during coal combustion. Therefore, this study investigated the influence of alkaline metals (Na_2_CO_3_, NaCl, and CaO) on the adsorption behavior of HMs (Pb, Cd, Cu, and Zn) onto quartz via tube furnace combustion experiments and the CASTEP module based on density functional theory (DFT). The results showed that the addition of Na_2_CO_3_ and NaCl was disadvantageous for the retention of HMs in the ash, particularly NaCl. With the increase in NaCl from 0 to 5 wt%, the immobilization efficiencies for Pb and Cd progressively declined from 33.99% and 37.78% to 9.89% and 12.04%, respectively. As the temperature increased from 800 °C to 1200 °C, the fixation rates of Cu, Zn, Pb, and Cd decreased by 19.96%, 27.75%, 23.35%, and 20.68%, respectively, when Na_2_CO_3_ was present in the coal. The results of the DFT demonstrated that the adsorption energy of alkali metals on quartz-α(001) surfaces was much greater than that of HMs, thus adversely affecting the adsorption of HMs. The adsorption energy of Na_2_O reached as high as −924.70 kJ/mol, while that of HMs was generally below −650 kJ/mol. This work contributed to a deeper understanding of the fate, migration, and transformation of HMs, thereby facilitating the mitigation of HMs release and subsequent associated ecological risks.

## 1. Introduction

In 2021, China’s overall energy consumption reached 5.24 billion tons of standard coal, with coal remaining the predominant source [1]. Although the composition of coal varies in different regions, they all present the risk of heavy metals (HMs) being released during combustion [2,3,4,5]. Semi-volatile HMs such as Pb, Cd, Cu, and Zn have garnered significant interest in recent years owing to their intricate transport mechanisms than those of high-volatile and non-volatile HMs [6,7,8]. HMs are not only a serious threat to human health, but also weaken the ability of catalysts to remove pollutants such as chlorinated volatile organic compounds and nitric oxides [9,10]. Therefore, it is essential to explore the control mechanism of HMs at high temperatures, in which mineral sorbents are promising in reducing the hazard of HMs [11,12].

Quartz (SiO_2_) is widely found in coal and could trap HMs vapors at high temperatures [13,14,15,16]. For example, Yu et al. [17] examined the adsorption capacity of the primary oxides found in coal on PbO at a temperature of 900 °C, and the results showed that the adsorption capacity of SiO_2_ on PbO was much higher than that of Fe_2_O_3_, Al_2_O_3_, CaO, and MgO, and the controlling effectiveness of SiO_2_ on Pb demonstrated an initial propensity to rise followed by a subsequent decline within the range of 700~1200 °C. Furthermore, the adsorption performance of SiO_2_ on Cd, Cu, and Zn has been widely reported by scholars [18,19,20]. However, these findings were constrained in scope, as they failed to account for the influence of other mineral-related components (e.g., alkaline metals) on the control of HMs release.

In recent years, numerous investigations have been conducted on the co-combustion of coal alongside alternative fuels, e.g., sludge, municipal waste, biomass, etc., which may increase the content of alkaline metals in the combustion system [21,22,23]. In addition, the high-alkaline coal in the Xinjiang Uygur Autonomous Region of China is often used in the process of coal blending combustion, due to the problems of slagging and ash deposition caused by its individual combustion, which would also introduce a certain amount of alkaline metals (Na and Ca), with the content of Ca being more significant [24,25,26].

Numerous studies have appeared related to the effect of alkaline Na and Ca species on the transport and transformation of HMs. For example, Liu et al. [27] investigated the migration and transformation characteristics of Pb, Zn, and Cu with the addition of NaCl to sludge using a tube furnace, and found that Pb largely existed in the form of PbCl_2_ (g) in the gas phase. Ca-based compounds also influence the behavior of HMs during combustion. Zha et al. [28] found that CaO promoted the evaporation of Cu from sludge in the range from 600 to 1100 °C, but increased the fixation rate of Zn at higher temperatures, and assumed that Ca could form eutectic silica-aluminate with Zn. It can be observed that existing studies primarily employ the approach of directly adding additives to the fuel. However, the inorganic components in fuel are often highly complex, which introduces numerous interfering factors, thereby increasing the uncertainty of experimental results. Accordingly, this study adopts a method that eliminates the interference of inherent minerals in coal, aiming to more rigorously elucidate the effect of variations in Na and Ca contents on the immobilization of HMs by quartz during coal combustion.

To better understand the effects and mechanisms of alkaline metals on HMs adsorption by SiO_2_, in this study, the raw coal was pre-treated using the HCl-HF method, which removed most of the minerals in the coal, and the impact on the primary organic framework of the unprocessed coal was negligible [29,30]. Then, SiO_2_, trace HMs, and alkaline metals were loaded into the demineralized coal at a certain content, so that the experimental coal was obtained. Accordingly, this study aimed to (1) quantify the influence of NaCl, Na_2_CO_3_, and CaO on the retention and chemical forms of Pb, Cd, Cu, and Zn during combustion of quartz-dominated coal; (2) elucidate the effects of temperature on HMs volatilization and stabilization; (3) analyze the microstructure and crystalline phase composition of coal fly ash; and (4) use density functional theory (DFT) methods to clarify the competitive adsorption mechanisms between alkaline metals and HMs on quartz surfaces.

## 2. Results and Discussion

### 2.1. HMs Behavior with Different Ratios of Alkaline Metals

#### 2.1.1. Fixation Rates of HMs

**Figure 1:**
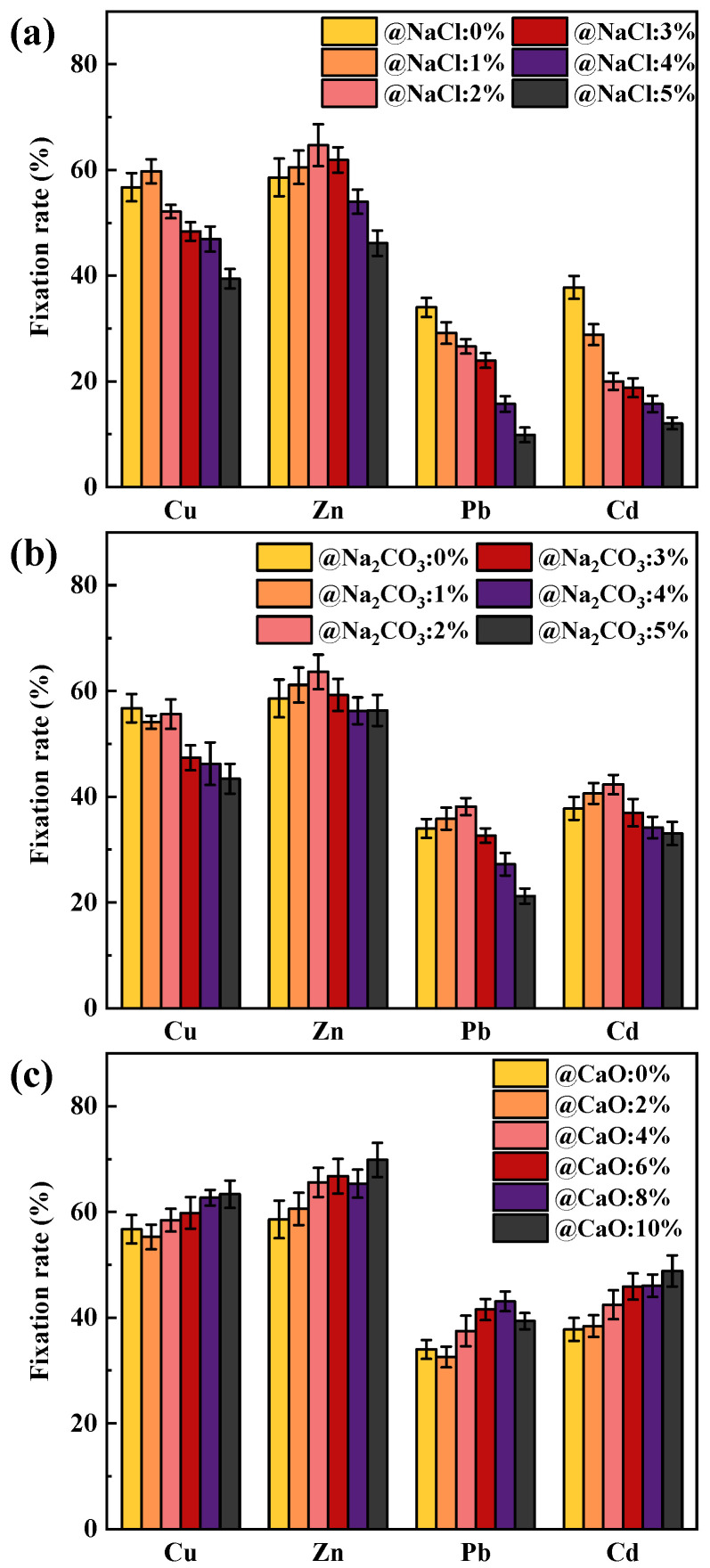
Effect of Na and Ca content on the immobilization rates of Cu, Zn, Pb, and Cd: (**a**) NaCl; (**b**) Na_2_CO_3_; (**c**) CaO.

The immobilization rates of HMs during the combustion of coal, primarily made up of SiO_2_ and loaded with alkaline metals at 1000 °C, are depicted in Figure 1. When coal was free of alkaline metals, the immobilization rates for Cu, Zn, Pb, and Cd were 56.72%, 58.60%, 33.99%, and 37.78%, respectively. Since PbO and CdO oxides exhibited lower boiling and melting points compared to CuO and ZnO, they were more easily released into the atmosphere at high temperatures, resulting in relatively lower fixation rates than Pb and Cd [31].

The effect of NaCl content on the HMs immobilization rate of SiO_2_-dominated coal is shown in Figure 1a. With the increase in NaCl addition, the fixation rates of Pb and Cd progressively decreased to 9.89% and 12.04% (@NaCl 5 wt%), respectively, whereas Cu and Zn displayed an elevated and then reduced trend. It was found that NaCl addition affected Pb and Cd to a greater extent than Cu and Zn. The decrease in the fixation rate of HMs was mainly attributed to the formation of low-melting-point chlorinated HMs and the competition between Na and HMs for the active sites. Chlorinated HMs may be formed through two reaction paths when SiO_2_, NaCl and oxidized HMs were co-present in the system [32]. On the one hand, NaCl and SiO_2_ react to form Na-containing silicates with the release of Cl_2_ or HCl, with the reaction formulae Equations (1) and (2) in the presence of oxygen and moisture. Then, Cl_2_ and HCl react with oxidized HMs to form chlorinated HMs in the reaction Equations (3) and (4), where M represents HMs. On the other hand, oxidized HMs can react with SiO_2_ to form HMs-containing silicates, Equations (5) and (6). Then, NaCl participated in the chlorination reaction, Equation (7). SiO_2_ could also chemisorb chlorinated HMs, with H_2_O and O_2_ playing a contributing role, Equations (8) and (9). However, Wang et al. [16] concluded that the inhibitory effect of SiO_2_ on the release of chlorinated HMs was not obvious in high-temperature adsorption experiments. Therefore, the fixation rate of HMs after NaCl addition demonstrated a more noticeable decreasing trend.

(1) $$\mathrm{NaCl}(g)\;+\;{\mathrm{SiO}}_{2}+\;O_{2}\left(g\right)\;\to \;{\mathrm{Na}}_{x}{\mathrm{Si}}_{y}O_{z}+{\mathrm{Cl}}_{2}\left(g\right)$$

(2) $$\mathrm{NaCl}(g)\;+\;{\mathrm{SiO}}_{2}+H_{2}O(g)\;\to \;{\mathrm{Na}}_{x}{\mathrm{Si}}_{y}O_{z}+\mathrm{HCl}(g)$$

(3) $${\mathrm{Cl}}_{2}(g)\;+\;\mathrm{MO}\;\to \;{\mathrm{MCl}}_{2}+O_{2}\left(g\right)$$

(4) $$\mathrm{HCl}(g)\;+\;\mathrm{MO}\;\to \;{\mathrm{MCl}}_{2}+H_{2}O(g)$$

(5) $$\mathrm{MO}+{\mathrm{SiO}}_{2}\;\to \;M_{x}{\mathrm{Si}}_{y}O_{z}$$

(6) $$\mathrm{MO}+{\mathrm{SiO}}_{2}+{\;O}_{2}(g)\;\to \;M_{x}{\mathrm{Si}}_{y}O_{z}$$

(7) $$\mathrm{NaCl}\;+\;M_{x}{\mathrm{Si}}_{y}O_{z}\;\to \;{\mathrm{Na}}_{x}{\mathrm{Si}}_{y}O_{z}+\;{\mathrm{MCl}}_{2}\left(g\right)$$

(8) $${\mathrm{MCl}}_{2}(g)\;+\;{\mathrm{SiO}}_{2}+H_{2}O(g)\;\to \;{\mathrm{MSiO}}_{3}+2\mathrm{HCl}(g)$$

(9) $$2x{\mathrm{MCl}}_{2}(g)+\mathrm{ySi}O_{2}+\;O_{2}(g)\;\to \;2\mathrm{xMO}\cdot \mathrm{ySi}O_{2}+{2\mathrm{xCl}}_{2}\left(g\right)$$

The effect of Na_2_CO_3_ content on the immobilization rate of HMs is shown in Figure 1b. The effect of Na_2_CO_3_ on the fixation rate of HMs was divided into two stages. The immobilization rate of Cu remained stable, while the fixation rates of Pb, Zn, and Cd were slightly increased when the Na_2_CO_3_ content was 1% and 2%. However, a decreasing trend in the immobilization rates of Zn, Pb, Cu, and Cd was observed at 3%, 4%, and 5% Na_2_CO_3_ content, which may result from the change in the physicochemical properties of the mineral after the capture of Na by the SiO_2_. Na_2_CO_3_ decomposed at high temperatures to gaseous Na_2_O, which reacted with SiO_2_ in a process that may proceed in the form of Equation (10) and Equation (11) to form low-melting Na-containing silicates [33]. Since the Na_2_CO_3_ content was relatively low, the mineral surface was in the early stages of melting, and capture of HMs was favored. However, the melting of the minerals intensified as the Na_2_CO_3_ content increased, at which point the minerals became completely deactivated, resulting in an increased release of HMs [34]. Therefore, it was the excessive Na_2_CO_3_ content (above 3%) that showed a more pronounced decrease in the fixation rate of HMs.

(10) $${\mathrm{Na}}_{2}O+3{\mathrm{SiO}}_{2}\;\to \;{\mathrm{Na}}_{2}{\mathrm{Si}}_{3}O_{7}$$

(11) $${\mathrm{Na}}_{2}O+2{\mathrm{SiO}}_{2}\;\to \;{\mathrm{Na}}_{2}{\mathrm{Si}}_{2}O_{5}$$

Figure 1c illustrates the impact of CaO content on the fixation rate of HMs. The fixation rate of HMs gradually increased with the addition of CaO content, indicating that the addition of CaO was favorable to immobilize more HMs in the coal ash. It is important to note that the reaction of CaO with SiO_2_ theoretically results in a reduction in active sites, reactions Equations (12) and (13), which hinder the fixation of HMs in the ash. The increase in the fixation rate of HMs may be due to the fact that SiO_2_, after chemisorption of CaO, does not seriously affect the subsequent adsorption of HMs, and CaO fixes the HMs by physical adsorption.

(12) $$2\mathrm{CaO}+{\mathrm{SiO}}_{2}\;\to \;{\mathrm{Ca}}_{2}{\mathrm{SiO}}_{4}$$

(13) $$\mathrm{CaO}+{\mathrm{SiO}}_{2}\;\to \;{\mathrm{CaSiO}}_{3}$$

#### 2.1.2. HMs Leaching Behavior

**Figure 2:**
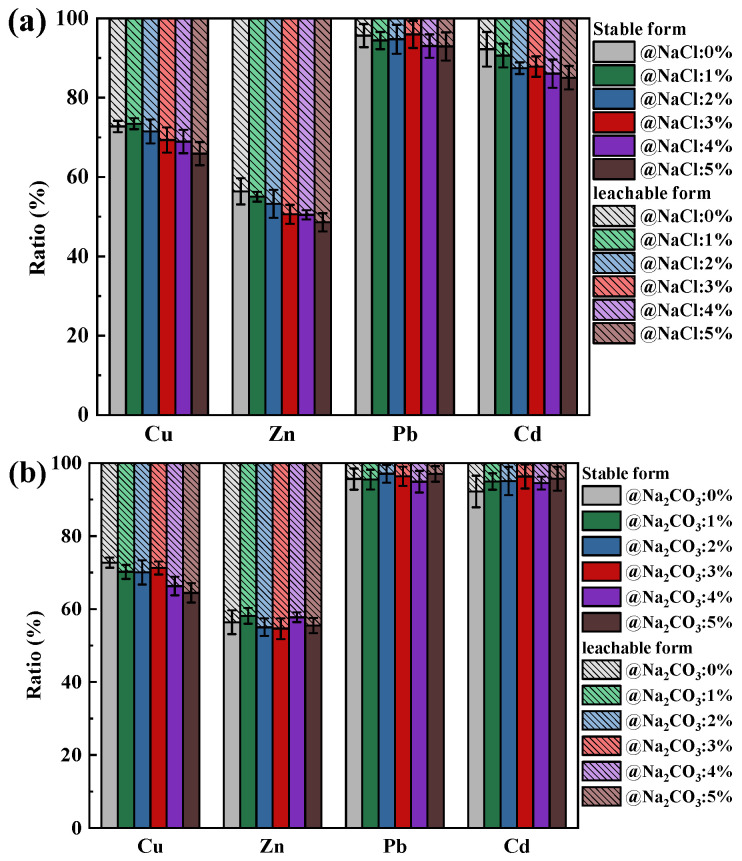

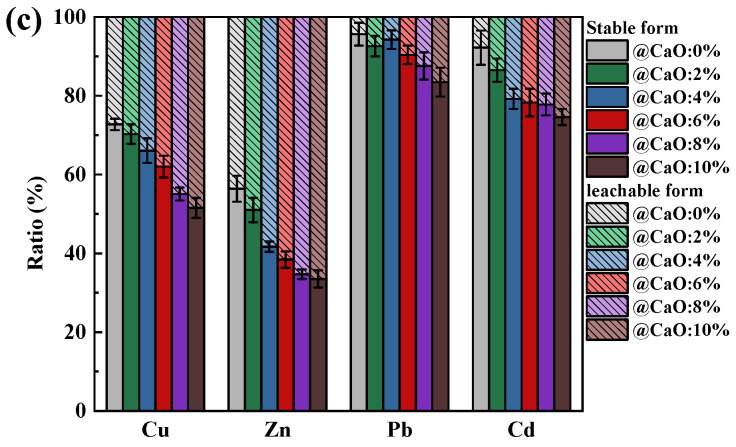
Effect of Na and Ca contents on the leaching behavior of Cu, Zn, Pb, and Cd in ash: (**a**) NaCl; (**b**) Na_2_CO_3_; (**c**) CaO.

The effect of changes in Na and Ca content on the leaching behavior of HMs forms in coal ash is shown in Figure 2. After the combustion of SiO_2_-dominated coal, the percentage of Cu, Zn, Pb, and Cd in the stable form was 72.73%, 56.39%, 95.66%, and 92.21%, respectively. Since the oxides of Pb and Cd were more readily released in gaseous form than Cu and Zn, most of the Pb and Cd retained in the ash was in the form of stable silicates, and thus a relatively large proportion of Pb and Cd was present in stable form.

It can be found that as the content of NaCl and Na_2_CO_3_ in SiO_2_-dominated coal increased, the HMs in the leachable state remained relatively stable (Figure 2a,b), indicating that SiO_2_ was still able to chemisorb most of the HMs under the influence of NaCl and Na_2_CO_3_. Especially for Pb and Cd, the proportion of them in a stable state was consistently above 80%, indicating that the risk of their leakage into the natural environment was relatively low. The percentage of HMs in the leachable state showed an increasing trend with increasing CaO addition (Figure 2c). Because of the physiosorption by CaO on HMs, the probability of chemical reaction of HMs with SiO_2_ was reduced, which heightens the risk of HM leakage from ash into the surrounding environment. However, the increase in HMs in the leachable state with CaO addition was more prominent compared to those with NaCl and Na_2_CO_3_. Therefore, additional attention needed to be paid to the exposure of HMs leached from coal ash.

### 2.2. HMs Behavior with Different Temperatures

#### 2.2.1. Fixation Rates of HMs

**Figure 3:**
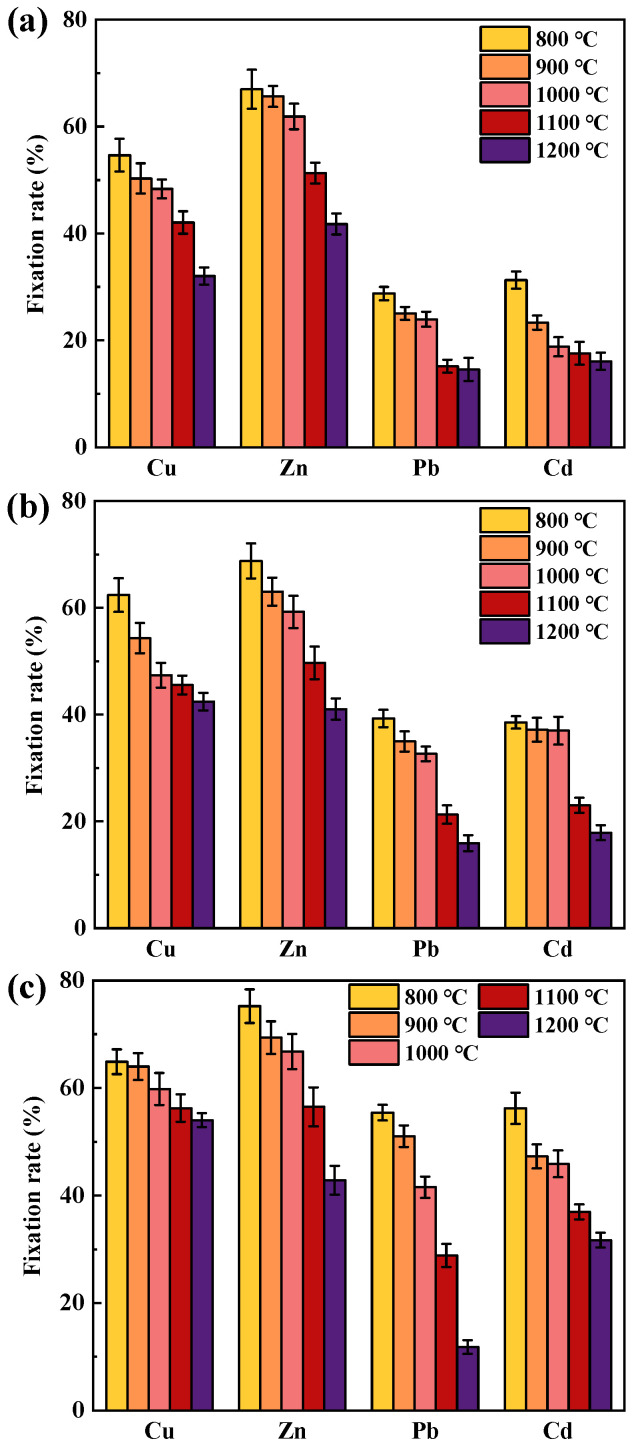
Temperature effects on the immobilization rates of Cu, Zn, Pb, and Cd: (**a**) NaCl existence; (**b**) Na_2_CO_3_ existence; (**c**) CaO existence.

The effect on the immobilization rate of HMs under different temperatures in SiO_2_-dominated coal is shown in Figure 3, with the contents of sodium chloride, sodium carbonate, and calcium oxide being 3 wt%, 3 wt%, and 6 wt%, respectively. The immobilization rate of HMs gradually decreased with increasing temperature when NaCl was present (Figure 3a). As the temperature increased from 800 °C to 1200 °C, the fixation rates of Cu, Zn, Pb, and Cd decreased by 22.60%, 25.23%, 14.21%, and 15.20%, respectively. The decrease in the immobilization rate of Cu, Zn, and Pb was above 1000 °C, which may be due to the increase in the content of chlorinated HMs at high temperatures and the increase in the diffusion rate of HMs. Compared to oxidized HMs, SiO_2_ was not effective in preventing the volatilization of chlorinated HMs [17,35], which were therefore more likely to be released in gaseous form.

The volatilization rate of HMs gradually increased with increasing temperature when Na_2_CO_3_ was present (Figure 3b). As the temperature increased from 800 °C to 1200 °C, the fixation rates of Cu, Zn, Pb, and Cd decreased by 19.96%, 27.75%, 23.35%, and 20.68%, respectively. At lower temperatures, the reaction between Na_2_O and SiO_2_ was kinetically restricted as the generation or diffusion of Na_2_O was restricted. At high temperatures, the chemical adsorption capacity of SiO_2_ to Na was enhanced, resulting in a reduction in active sites on the mineral surface. In addition, the higher the temperature, the greater the volatility of HMs, and the lower the ability of SiO_2_ to control HMs, which were the reasons for the decrease in fixation rate. For example, Yu et al. [17] carried out high-temperature adsorption of PbO on SiO_2_ at 700~1200 °C, and found that the immobilization rate of Pb gradually decreased after 900 °C. Compared with loading NaCl, a relatively higher immobilization rate was observed in loaded Na_2_CO_3_. This may be due to the lack of Cl interference in the combustion system, so most HMs exist in the form of oxides. Compared with chlorinated HMs, their melting and boiling points were lower and easier to retain in coal ash.

The effect of temperature on the volatilization rate of HMs when the SiO_2_-dominated coal contains CaO is shown in Figure 3c. At 800 °C, the immobilization rates of Cu, Zn, Pb, and Cd were 64.87%, 75.21%, 55.43%, and 56.23%, respectively, which were greater than those in the presence of Na compounds, suggesting that CaO inhibited the volatilization of HMs. As the combustion temperature increased, the fixation rate of HMs gradually decreased, with the decrease in Pb was the largest. When the temperature was 1200 °C, the fixation rate of Pb was only 11.80%. In addition to the high volatility of HMs at high temperatures, the decrease in HMs fixation rate was also related to the transformation of CaO at high temperatures. Ca existed in the form of CaCO_3_ during coal combustion, and decomposition to CaO at about 750 °C. The obtained products had a high adsorption pore volume and pore specific surface area distribution of about 800 °C. Increasing the temperature may lead to sintering and agglomeration of grains, which reduces the adsorption capacity of CaO to HMs [36].

#### 2.2.2. HMs Leaching Behavior

**Figure 4:**
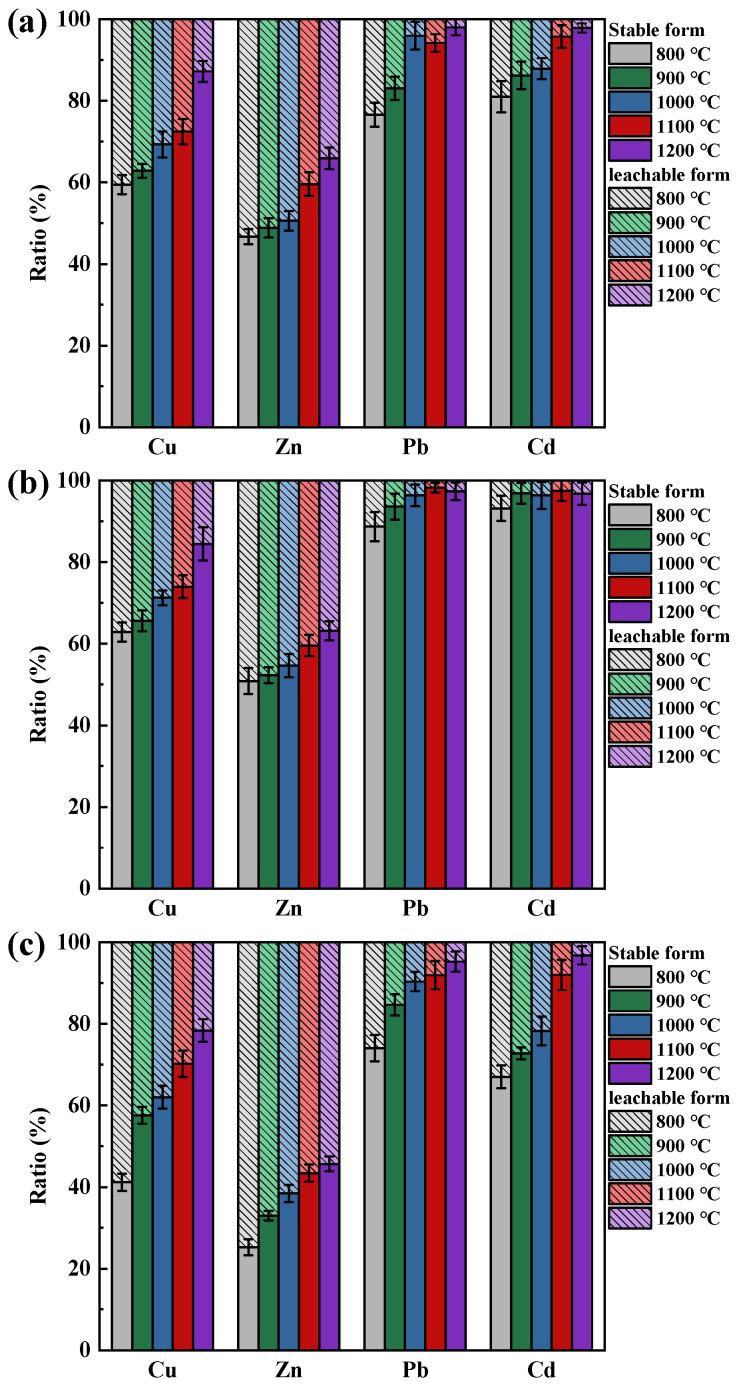
Temperature effects on the leaching behavior of Cu, Zn, Pb, and Cd in ash: (**a**) NaCl existence; (**b**) Na_2_CO_3_ existence; (**c**) CaO existence.

The effect on the leaching behavior of HMs due to temperature is shown in Figure 4. It can be found that with an increase in temperature, the proportion of stable form HMs progressively increases, thereby reducing the risk of HMs leaching. Among these, Zn faced the greatest leaching risk. The percentage of Zn in the leachable form after combustion at 800 °C of coal loaded with NaCl, Na_2_CO_3_, and CaO was 53.33%, 49.15%, and 74.76%, respectively. As the temperature reached 1200 °C, although the leachable form of Zn decreased to 34.08%, 36.81%, and 54.33%, a considerable part of Zn was still easily leached under weak acid conditions.

As the combustion temperature increased, Pb and Cd were more likely to exist in the coal ash in stable form. For samples loaded with NaCl and Na_2_CO_3_, the percentage of stable forms Pb and Cd remained above 80% and 90%, respectively, after temperatures greater than 900 °C. When the coal was loaded with CaO, the percentage of stable forms of Pb and Cd remained above 90% at temperatures greater than 1100 °C. This was probably due to the strong volatility of Cd and Pb at high temperatures. SiO_2_ does not effectively inhibit their diffusion, and most of the Pb and Cd retained in the ash were stably fixed in the form of chemical adsorption.

### 2.3. Characterization Analysis

#### 2.3.1. SEM Analysis

**Figure 5:**
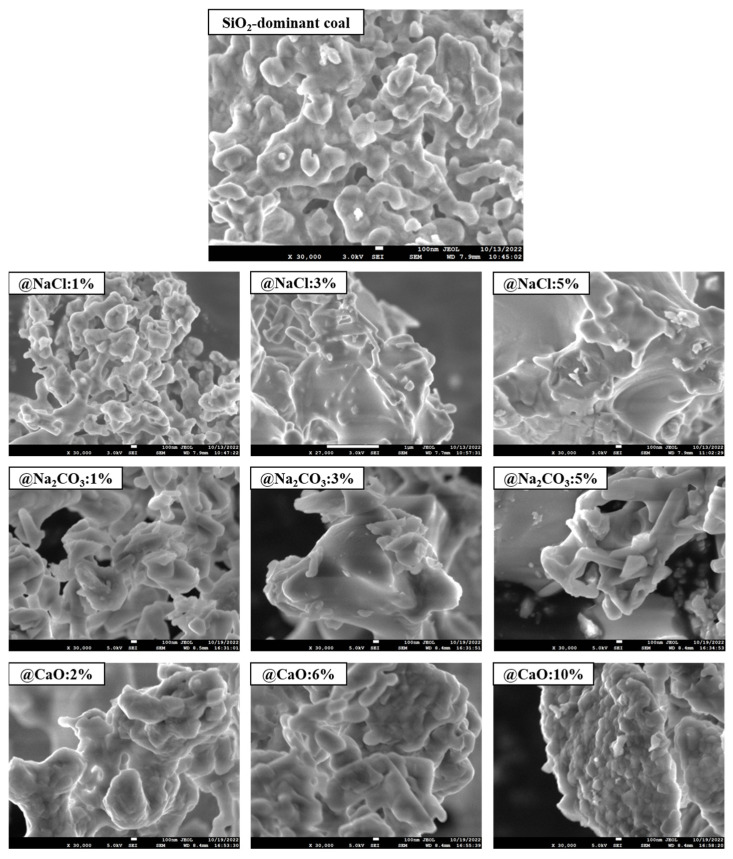
Morphology of coal ash from SiO_2_-dominated coal loaded with alkaline metals at 1000 °C.

The effect of alkaline metals addition on the micro-morphology of the coal ash surface at 1000 °C is illustrated in Figure 5. It can be observed that the pore structure of SiO_2_-dominated coal ash was abundant, indicating that the metal vapors could freely enter the interior of the mineral with strong reactivity. As the NaCl and Na_2_CO_3_ content increased, the melting of the SiO_2_ surface grew more severe. When the content of NaCl and Na_2_CO_3_ increased to 5%, the surface became quite smooth, indicating that the addition of Na formed many of the lower-melting-point minerals. These minerals were wrapped on the SiO_2_ surface in liquid form at high temperatures, which reduced the active sites of SiO_2_. As the CaO content increased, the mineral gradually transformed into larger particles with the occurrence of a slight melting phenomenon, which may be related to the formation of wollastonite (melting point 1540 °C), a reaction between CaO and SiO_2_ [37].

#### 2.3.2. XRD Analysis

**Figure 6:**
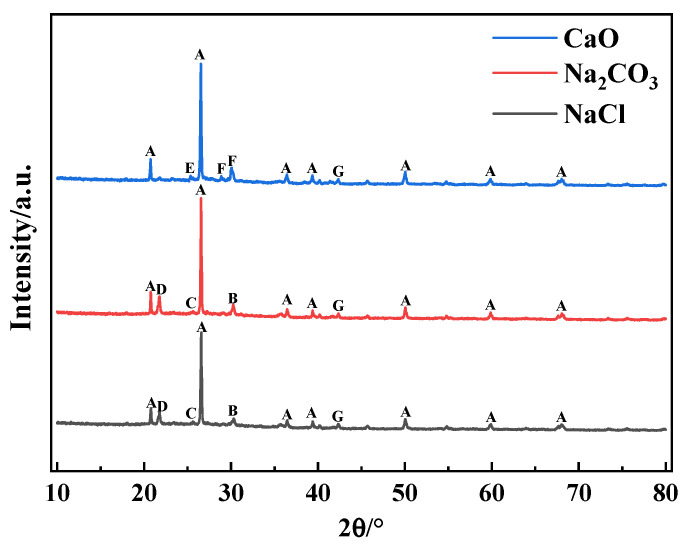
XRD patterns of coal ash after combustion of SiO_2_-dominated coal loaded with alkaline metals at 1000 °C. A—quartz [SiO_2_]; B—Sodium zinc silicate [Na_2_ZnSiO_4_]; C—Lead silicate [Pb_3_Si_2_O_7_]; D—Zinc silicate [Zn_2_SiO_4_]; E—Alamosite [PbSiO_3_]; F—Wollastonite [CaSiO_3_]; G—Zinc oxide [ZnO].

XRD analyses of SiO_2_-dominated coal loaded with constant heavy and alkaline metals after combustion are demonstrated in Figure 6. When the coal contained NaCl and Na_2_CO_3_, HMs-containing crystalline phases, including sodium zinc silicate, lead silicate, zinc silicate, and zinc oxide, were detected in the ash, suggesting that SiO_2_ was capable of chemisorbing Pb and Zn at high temperatures, ultimately immobilizing the HMs in the form of silicates. However, no Cd-containing and Cu-containing substances were detected in the ash, which may be due to the presence of related compounds in an amorphous state. In addition, the presence of alamosite was detected in the ash when CaO was present, whereas the product of Pb was lead silicate in the presence of NaCl and Na_2_CO_3_, suggesting that the species of alkaline metals affected the production of the reaction between HMs and SiO_2_.

### 2.4. Computational Results and Discussion

#### 2.4.1. HMs Oxides and Chlorides Adsorption on SiO_2_(001) Surface

The adsorption configurations of HMs oxides and chlorides on the SiO_2_(001) surface are presented in Table A1. The adsorption energies of HMs on the SiO_2_(001) surface are shown in Table 1. The adsorption energies of CuO, ZnO, PbO, and CdO were −620.03 kJ/mol, −505.15 kJ/mol, −378.10 kJ/mol, and −551.94 kJ/mol, respectively, while those of CuCl_2_, ZnCl_2_, PbCl_2_, and CdCl_2_ were −216.65 kJ/mol, −124.07 kJ/mol, −152.64 kJ/mol, and −144.61 kJ/mol, respectively. These results indicated that SiO_2_ was chemisorbed for both oxidized and chlorinated HMs.

The Mulliken bond population analysis of HMs molecules and their interacting atoms after the HMs adsorption on the SiO_2_(001) surface is provided in Table A2. Higher population indicated a stronger interaction force between the atoms [38]. It can be found from Table A2 (a) that the bond population between O and Si(II) atoms was larger than that between heavy metal atoms and Si(IV) atoms, indicating that the O atoms were the main factor for the adsorption of oxidized HMs on the SiO_2_(001) surface. From Table A2 (b), it could be revealed that the bond population of heavy metal atoms and Si(IV) atoms was relatively larger, indicating that the interaction of Si(IV) with heavy metal atoms dominated the occurrence of chlorinated HMs adsorption.

#### 2.4.2. Alkaline Metals Adsorption on SiO_2_

The optimized structures of NaCl, Na_2_O, and CaO adsorption on the SiO_2_(001) surface are shown in Table A3. The adsorption energies of NaCl, Na_2_O, and CaO on the SiO_2_(001) surface were −494.31 kJ/mol, −924.70 kJ/mol, and −587.13 kJ/mol, respectively, indicating that SiO_2_ has better adsorption properties for alkaline metals than HMs. It can be found that the Na atom in the NaCl molecule was spatially close to the Si(IV) atom and the Cl atom was close to the Si(II) atom after adsorption stabilization. O atoms (from Na_2_O and CaO) were closer to Si(IV) atoms. Since Si(II) and Si(IV) were also active sites for the adsorption of HMs, it can be speculated that the adsorption of alkaline metals on the SiO_2_(001) surface will affect the subsequent adsorption of HMs.

#### 2.4.3. Influence of Alkaline Metal-SiO_2_ Interactions on the Adsorption of HMs

The optimized structures of the HMs adsorption on the surface of NaCl/Na_2_O/CaO-SiO_2_ (001) are displayed in Table A4. From Table 1, it can be found that pre-adsorption of alkaline metals caused the adsorption energy of HMs to decrease to different degrees.

Analysis of the Mulliken bonding numbers of HMs molecules and their interacting atoms after adsorption of HMs on the surface of NaCl/Na_2_O/CaO-SiO_2_(001) is listed in Table A5. After HMs adsorption on NaCl-SiO_2_(001) surface, the bond population between Pb and Si(IV) atoms was 0.35, which was less than that of Cu (0.63), Zn (0.63), and Cd (0.56) atoms. After HMs adsorption on the Na_2_O-SiO_2_(001) surface, the bond populations of Cu, Zn, Pb, and Cd atoms with Si (III) atoms were 0.87, 0.7, 0.14, and 0.55, respectively. The above results indicate that the interaction between the Pb atom and substrate was relatively weak compared to Cd, Zn, and Cu, when the SiO_2_(001) surface was pre-adsorbed with NaCl and Na_2_O, which is in agreement with the experimental study. The interaction between HMs and alkali species was dominated by competition for Si-O active sites on the quartz surface. No direct electron transfer or redox transition between heavy-metal ions and alkali ions was implied by the DFT results.

Comparison of the co-adsorption energy calculation results and combustion test results found that they were not in good agreement. When the NaCl and Na_2_O were pre-adsorbed on the SiO_2_(001) surface, the matrix still exhibited a relatively strong chemisorption capacity for the HMs, which was not consistent with the experimental results that the fixation rate of HMs was significantly reduced by the addition of NaCl and Na_2_CO_3_. However, it should be noted that SiO_2_(001) surface had significant adsorption capacity for NaCl and Na_2_O with adsorption energies of −494.31 kJ/mol and −924.70 kJ/mol, respectively, which were much larger than the HMs. Considering that the content of alkali metals was well above the HMs, alkali metals would be the earliest to occupy the active sites on the SiO_2_(001) surface during the combustion process, thus leading to a reduced possibility of co-adsorption of alkali and HMs [39]. Therefore, a significant decrease in the immobilization rate of HMs by the presence of NaCl and Na_2_CO_3_ additions was observed in the experiments. It should be clarified that the DFT model reveals intrinsic thermodynamic trends, whereas experimental behavior is dominated by melt-induced kinetic effects [40].

## 3. Materials and Methods

### 3.1. Preparation of Demineralized Coal

The coal sourced from Shaer Lake, Xinjiang Province, China, was chosen as the feedstock for the preparation of demineralized coal (DEMc). The unprocessed coal was subjected to crushing and sieving to achieve a particle size of 100–120 μm. The HF-HCl method was employed for the preliminary treatment of raw coal [30,41]. First, the raw coal was mixed with HCl (37%) solution at 1 g:3 mL and placed on a magnetic stirrer for 24 h. The material was subsequently subjected to filtration and rinsed in ultra-pure water until achieving a pH of roughly 7. Washing of HCl-washed coal by rinsing with HF solution, the coal was mixed with HF (40%) solution at 1 g:3 mL and placed on a magnetic stirrer for 24 h [42]. Ultimately, the coal subjected to double acid washing was filtered and rinsed until the filtrate reached a neutral pH, followed by drying in a vacuum oven at 105 °C for 12 h. The proximate and ultimate analyses for both the raw and DEMc samples are presented in Table 2. The measured ash content of DEMc was 0.88 wt%, demonstrating that the majority of mineral constituents had been effectively eliminated from primary coal.

### 3.2. Preparation of SiO_2_-Dominated Coal

**Figure A1:**
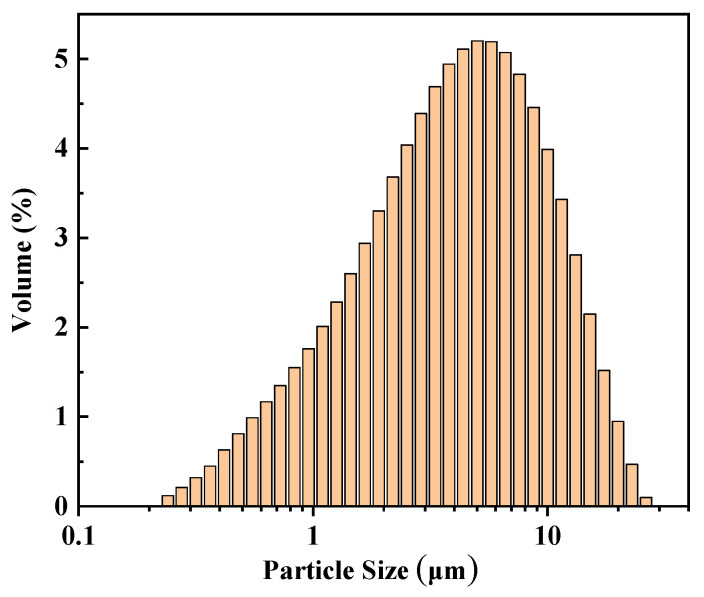
Particle size distribution of SiO_2_.

The chemical composition and structural properties of SiO_2_ are shown in Table 3 and Table 4, respectively. The particle size of SiO_2_ was tested using a Malvern Mastersizer 2000 (Malvern Panalytical, Malvern, Worcestershire, UK), and the results are shown in Figure A1.

Initially, DEMc and SiO_2_ (20 wt%) were dissolved in 10 g of the resultant mix in 15 mL deionized water and 15 mL anhydrous ethanol, stirring for a duration of 24 h. The mass percentage of SiO_2_ was determined based on the reference range of coal ash content [43,44]. Subsequently, the mixture was subjected to drying at 105 °C to yield SiO_2_-dominated coal. The SiO_2_-dominated coal was then blended with NaCl, Na_2_CO_3_, and CaO in specific proportions. The concentrations of Na and Ca were set based on the compositional characteristics of high-alkali coal [45]. Specifically, NaCl and Na_2_CO_3_ were added at 1~5 wt%, while CaO was set at 2~10 wt%. Finally, the samples were loaded with HMs acetate (Cu 200 mg/kg; Zn 200 mg/kg; Pb 500 mg/kg; Cd 500 mg/kg). The HMs loading was determined with reference to the experimental protocol described by Cai et al. [46]. The concentrations of HMs in the SiO_2_-dominated coal were documented in Table 5.

### 3.3. Combustion Experiment

**Figure A2:**
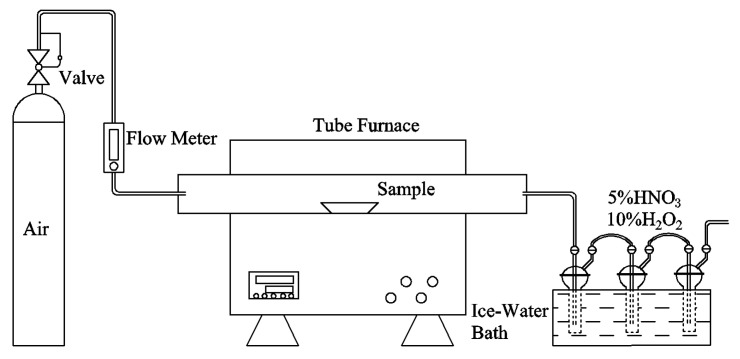
High-temperature horizontal tube furnace.

The combustion experiments were conducted using a horizontal tube furnace, model SGL-1700, from Shanghai Jujing Precision Instrument Manufacturing Co., Ltd., Shanghai, China, as depicted in Figure A2. This tube furnace was engineered in a closed chamber, utilizing corundum tube of 30 mm diameter and 1000 mm length. Each test involved placing 2.0 ± 0.01 g of the sample into a corundum crucible, which was then inserted into the furnace. When investigating the effect of alkaline metal content variation on the migration and transformation of HMs, the combustion temperature was fixed at 1000 °C. Additionally, a certain amount of alkaline metals was added to the samples, which were then heated to 800 °C, 900 °C, 1000 °C, 1100 °C, and 1200 °C, respectively, to study the influence of temperature changes on HMs. The combustion was carried out under an air atmosphere with a flow rate of 600 mL/min and a heating rate of 15 °C/min, and the combustion lasted for 1 h at the designated temperature. After the combustion tests, the ash remaining in the crucibles was collected for subsequent analysis. To reduce experimental errors, three parallel sets of tests were carried out.

### 3.4. HMs Detection

The total amount of HMs in the samples was obtained using ICP-OES (ICAP 7000, Thermo Fisher, Worcester, MA, USA). A total of 100 ± 0.5 mg of each ash sample was digested in a microwave digestion system (HANON TANK, Jinan, Shandong province, China) using a mixed acid solution consisting of 8 mL of 65% HNO_3_, 2 mL of 30% H_2_O_2_, and 2 mL of 40% HF. The digestion process was conducted at 180 °C for 40 min, ensuring complete dissolution of the coal ash. HMs leaching from the coal ash was performed via the Toxicity Characteristic Leaching Procedure (TCLP). For this, an acetate buffer (pH 2.88) was added to each sample at a liquid-to-solid ratio of 20:1, followed by mixing on a rotary shaker for 18 h. Subsequent to shaking, the samples were filtered to collect the HMs leachate, while the residual solids were subjected to complete digestion treatment [47,48]. The stable form indicated that HMs were incorporated into the mineral lattice in the form of silicates, which were not prone to leaching into the natural environment. In contrast, the leachable form referred to HMs physically adsorbed onto the mineral surface, which could be readily leached in acidic aqueous solutions. The distribution of HMs was computed using the following equation:

(14) $$\eta \;=\;\frac{M_{A}}{M_{C}}\;\times \;100\%$$

(15) $$R_{L\;}=\frac{M_{L}}{M_{A}}\;\times \;100\%$$

(16) $$R_{s}=\frac{M_{S}}{M_{A}}\;\times \;100\%$$

where M_C_ represents mass of the HMs within the coal, M_A_ denotes the mass of the HMs within the coal ash, and M_L_ and M_S_ refer to the masses of HMs leached and stabilized within the coal ash, respectively. η signifies the percentage of HMs immobilization, while R_L_ and R_S_ indicate the proportions of leached and stabilized forms of HMs within the coal ash, respectively.

### 3.5. Sample Characterization

The micro-morphology of coal ash was tested using an electron scanning electron microscope (SEM), model JSM-7610F, from Nippon Electron Co, Japan. The mineral composition of coal ash was determined by X-Ray Powder Diffraction (XRD, Rigaku’s Smartlab, Wako, Japan). The test parameters were set to Cu target ray, working power set to 4 kW, and the sample was scanned between 2θ values of 10° and 80° at a step size of 10°. It must be declared that the coal ash tested by XRD was obtained by burning coal with a constant HMs content. The content of NaCl, Na_2_CO_3_, and CaO used in the experiment was 2 wt%, and the content of acetates containing Cu, Zn, Pb, and Cd was 2 wt%, respectively.

### 3.6. DFT Computation Details

The DFT computation was performed using the CASTEP (Cambridge Sequential Total Energy Package) module within the Materials Studio 2021 Software. The DFT computation was conducted using a plane-wave pseudopotential framework, applying the generalized gradient approximation according to Perdew–Burke–Ernzerhof for the exchange function [49]. It should be noted that dispersion correction and electron U addition have not been considered, which may lead to deviations in the value of adsorption energy [50,51]. The OTFG ultrasoft pseudopotential to model the interactions between real ions and valence electrons [52]. The geometric optimization was executed using the Broyden Fletcher Goldfarb Shanno (BFGS) algorithm [53]. To ensure the accuracy of the simulation calculation results, the relevant parameters of the optimization calculation were set as follows: cutoff energy was set to 500 eV; the optimal convergence criterion for total energy difference was set to 1.0 × 10^−5^ eV/atom; 0.03 eV/Å for the maximum force between atoms; stress was less than 0.05 GPa; and 1.0 × 10^−3^ Å for maximum displacement. The self-consistent field (SCF) convergence accuracy was 5.0 × 10^−7^ eV/atom.

**Figure 7:**
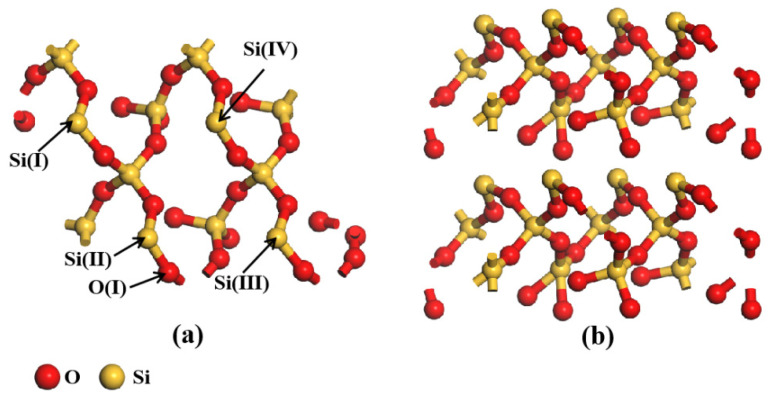
The structure diagram of SiO_2_: (**a**) top view; (**b**) side view.

The substrate was chosen using the quartz-alpha structure that was supplied in the Materials Studio 2021 Software. The (001) crystalline surface of SiO_2_ with the strongest adsorption capacity for HMs was selected for this paper, a reference to the study of Yu et al. [17]. The structure of SiO_2_ unit cells was first optimized and then faceted. The configuration was then supercell-built (2 × 1 × 1), and a vacuum layer with a thickness of 20 Å was created above the (001) surface. The final SiO_2_ structure obtained is shown in Figure 7. Some of the atoms have been named to clearly express the bonding of HMs after adsorption on the surface.

**Figure 8:**
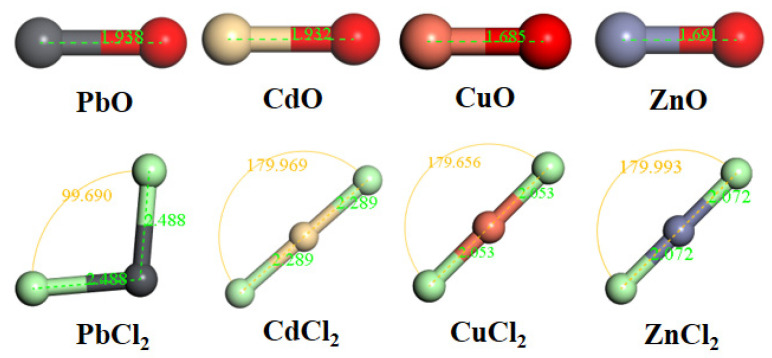
The optimized configurations of oxidized and chlorinated HMs.

The HMs chlorides and oxides were selected for the study, as heavy metal acetates undergo chemical conversion at high temperatures. The optimized configurations of HMs are shown in Figure 8. As Na_2_CO_3_ decomposed to Na_2_O and participated in reactions at 785 °C, Na_2_O was selected for adsorption by SiO_2_ [54,55]. To explore the influence of NaCl, Na_2_O, and CaO on HMs adsorption to SiO_2_(001) surface, the co-adsorption of HMs oxide with Na_2_O, HMs oxide with CaO, and HMs chloride with NaCl was examined accordingly. The adsorption energy (E_ad_, kJ/mol) was computed based on Equation (17).

E_ad_ = E_A+S_ − E_A_ − E_S_ (17)

where E_A_, E_S_, and E_A+S_ represent the energies of the adsorbate, substrate, and adsorption system, respectively.

## 4. Conclusions

In this research, the impact of alkaline metals (Na_2_CO_3_, NaCl, and CaO) on the adsorption capacity of quartz concerning Copper, Zinc, Lead, and Cadmium were examined during coal combustion.

The inclusion of NaCl and Na_2_CO_3_ negatively influenced the preservation of HMs within the ash, with NaCl being more significant in contributing to the release of HMs. As the addition of NaCl increased to 5 wt%, the fixation rates of Cu, Zn, Pb, and Cd decreased by 17.28%, 12.45%, 24.11%, and 25.73%, respectively. The addition of CaO favored the retention of HMs in the ash. An increase in the combustion temperature will increase the volatilization of HMs. Pb and Cd within the ash were more easily leached into the natural environment compared with Cu and Zn. After CaO addition, the ecological risk of Cu, Zn, Pb, and Cd leaching from the ash increased by 21.20%, 22.91%, 12.20%, and 17.63%, respectively. Elevated combustion temperatures reduced the proportion of leachable HMs in the ash. The DFT results indicated that the quartz-α(001) surface was capable of achieving chemisorption of chlorinated and oxidized HMs. The adsorption energy for oxidized HMs was higher than chlorinated HMs. After the alkaline metals were pre-adsorbed on the quartz-α(001) surface, the substrate still had a certain degree of chemisorption capacity for HMs.

The combined experimental and DFT study clarified the competitive adsorption mechanism between HMs and alkali metals on the quartz surface. This work provided practical guidance for optimizing the blended combustion of high-alkaline coal as well as the co-combustion systems involving coal and alkali metal-containing wastes. These findings helped improve ash management strategies and mitigate the environmental risks posed by HMs emissions.

## Figures and Tables

**Table 1:** HMs oxides and chlorides adsorption energies on the SiO_2_(001) surface and in the existence of NaCl, Na_2_O, and CaO (kJ/mol).

HMs	Oxides	Chlorides	Chlorides (NaCl Existence)	Oxides (Na_2_O Existence)	Oxides (CaO Existence)
Cu	−650.03	−216.65	−222.82	−583.16	−629.81
Zn	−505.15	−124.07	−95.01	−417.96	−521.75
Pb	−378.10	−152.64	−113.76	−211.71	−290.67
Cd	−551.94	−144.61	−78.12	−532.17	−515.49

**Table 2:** The proximate and ultimate analysis of raw and demineralized coal.

Coal Sample	Proximate Analysis (wt.%, Dry Basis)				Ultimate Analysis (wt.%, Dry and Ash-Free Basis)				
	M	A	V	FC	C	H	O	N	S
Raw coal	4.62	12.23	37.86	45.29	67.35	3.62	27.23	1.01	0.79
Demineralized coal	1.65	0.88	38.75	58.72	68.56	3.98	26.39	0.92	0.15

M: moisture; FC: fixed carbon; V: volatiles matter; A: ash content.

**Table 3:** Chemical composition of SiO_2_.

**Composition** **(wt%)**	**SiO_2_**	**Al_2_O_3_**	**K_2_O**	**MgO**	**CaO**	**Other**
	97.22	0.50	0.21	0.52	0.24	1.31

**Table 4:** Structural properties of SiO_2_.

**Structural Properties**	**Surface Area/(m^2^/g)**	**Pore Diameter/nm**	**Pore Volume/(×10^−2^ cm^3^/g)**
	3.50	7.03	0.14

**Table 5:** The HMs concentrations of the HMs supplements and SiO_2_-dominated coal.

Heavy Metals (mg/kg)	Cu	Zn	Pb	Cd
Supplement	200	200	500	500
SiO_2_-dominated coal	202.32	198.21	501.85	503.24

## Data Availability

The authors do not have permission to share data.

## Abbreviations

The following abbreviations are used in this manuscript:

HMs	Heavy metals
DFT	Density functional theory
DEMc	demineralized coal

## Appendix A

The particle size distribution of SiO_2_ is shown in Figure A1. The schematic diagram of the high-temperature horizontal tube furnace is shown in Figure A2. HMs oxides and chlorides optimize configurations for adsorption on SiO_2_(001) surfaces are summarized in Table A1. Mulliken bond population analysis of oxidized and chlorinated HMs adsorption on SiO_2_ is summarized in Table A2. The optimized configurations of NaCl, Na_2_O, and CaO adsorption over SiO_2_(001) surface are summarized in Table A3. The optimized structures of the HMs adsorption on the surface of NaCl/Na_2_O/CaO-SiO_2_(001) are summarized in Table A4. The Mulliken bond population analysis of HMs adsorption on NaCl/Na_2_O/CaO-SiO_2_ surface is summarized in Table A5.

**Table A1:** HMs oxides and chlorides optimize configurations for adsorption on SiO_2_(001) surfaces.

HMs		Cu	Zn	Pb	Cd
Oxides HMs adsorption structures	Top view				
	Side view				
Chlorides HMs adsorption structures	Top view				
	Side view				

Notes: .

**Table A2:** Mulliken bond population analysis of oxidized and chlorinated HMs adsorption on SiO_2_: (a) oxidized HMs; (b) chlorinated HMs.

(**a**)											
**CuO**			**ZnO**			**PbO**			**CdO**		
**Bond**	**D(Å)**	**P**	**Bond**	**D(Å)**	**P**	**Bond**	**D(Å)**	**P**	**Bond**	**D(Å)**	**P**
Cu-O	1.928	0.24	Zn-O	1.958	0.21	Pb-O	2.319	0.13	Cd-O	2.278	0.12
Cu-Si(IV)	2.201	0.59	Zn-Si(IV)	2.473	0.48	Pb-Si(IV)	2.872	0.46	Cd-Si(IV)	2.719	0.38
O-Si(II)	1.562	0.79	O-Si(II)	1.566	0.73	O-Si(II)	1.563	0.74	O-Si(II)	1.553	0.79
(**b**)											
**CuCl_2_**			**ZnCl_2_**			**PbCl_2_**			**CdCl_2_**		
**Bond**	**D(Å)**	**P**	**Bond**	**D(Å)**	**P**	**Bond**	**D(Å)**	**P**	**Bond**	**D(Å)**	**P**
Cu-Cl(I)	2.474	0.06	Zn-Cl(I)	2.434	0.16	Pb-Cl(I)	2.922	0.07	Cd-Cl(I)	2.715	0.10
Cu-Cl(II)	2.093	0.46	Zn-Cl(II)	2.107	0.61	Pb-Cl(II)	2.483	0.31	Cd-Cl(II)	2.319	0.51
Cu-Si(IV)	2.203	0.54	Zn-Si(IV)	2.355	0.61	Pb-Si(IV)	2.941	0.36	Cd-Si(IV)	2.522	0.56
Cl(I)-Si(I)	2.102	0.44	Cl(I)-Si(I)	2.186	0.34	Cl(I)-Si(I)	2.166	0.32	Cl(I)-Si(I)	2.160	0.36

Notes: D(Å)-Bond length (Å); P-Population; Cl(I) and Cl(II) represent the top and bottom Cl atoms (from chlorinated HMs) in the top view (Table A1), respectively.

**Table A3:** The optimized configurations of NaCl, Na_2_O, and CaO adsorption over SiO_2_(001) surface.

Alkaline Metals	NaCl	Na_2_O	CaO
Top view			
Side view			

Notes: .

**Table A4:** The optimized structures of the HMs adsorption on the surface of NaCl/Na_2_O/CaO-SiO_2_(001).

	HMs	Cu	Zn	Pb	Cd
Alkaline Metals					
NaCl	Top view				
	Side view				
Na_2_O	Top view				
	Side view				
CaO	Top view				
	Side view				

Notes: .

**Table A5:** Mulliken bond population analysis of HMs adsorption on NaCl/Na_2_O/CaO-SiO_2_ surface: (a) chlorinated HMs adsorption on the NaCl-SiO_2_ surface; (b) oxidized HMs adsorption on the Na_2_O-SiO_2_ surface; (c) oxidized HMs adsorption on the CaO-SiO_2_ surface.

**(a)**											
**CuCl_2_**			**ZnCl_2_**			**PbCl_2_**			**CdCl_2_**		
**Bond**	**D(Å)**	**P**	**Bond**	**D(Å)**	**P**	**Bond**	**D(Å)**	**P**	**Bond**	**D(Å)**	**P**
Cu-Cl(Ⅰ)	2.906	0.03	Zn-Cl(Ⅰ)	2.229	0.42	Pb-Cl(Ⅰ)	2.639	0.22	Cd-Cl(Ⅰ)	2.484	0.33
Cu-Cl(Ⅱ)	2.132	0.47	Zn-Cl(Ⅱ)	2.183	0.53	Pb-Cl(Ⅱ)	2.573	0.27	Cd-Cl(Ⅱ)	2.376	0.45
Cu-Si(Ⅳ)	2.154	0.63	Zn-Si(Ⅳ)	2.335	0.63	Pb-Si(Ⅳ)	2.909	0.35	Cd-Si(Ⅳ)	2.503	0.56
Cl(Ⅰ)-Si(Ⅰ)	2.143	0.37	Cl(Ⅰ)-Na	2.789	0.03	Cl(Ⅰ)-Na	2.627	0.10	Cl(Ⅰ)-Na	2.685	0.06
Cl(Ⅱ)-Na	2.558	0.08	Cl(Ⅱ)-Na	2.759	0.03	Cl(Ⅱ)-Na	2.708	0.08	Cl(Ⅱ)-Na	2.875	0.05
(**b**)											
**CuO**			**ZnO**			**PbO**			**CdO**		
**Bond**	**D(Å)**	**P**	**Bond**	**D(Å)**	**P**	**Bond**	**D(Å)**	**P**	**Bond**	**D(Å)**	**P**
Cu-O	1.852	0.40	Zn-O	1.918	0.32	Pb-O	2.193	0.20	Cd-O	3.357	-
Cu-Si(Ⅱ)	2.877	-	Zn-Si(Ⅱ)	3.126	-	Pb-O(Ⅰ)	2.469	0.05	Cd-Si(Ⅱ)	2.631	0.53
Cu-Si(Ⅲ)	2.161	0.87	Zn-Si(Ⅲ)	2.463	0.70	Pb-Si(Ⅲ)	3.532	0.14	Cd-Si(Ⅲ)	2.722	0.55
O-Na(Ⅱ)	2.260	-	O-Na(Ⅱ)	2.261	-	O-Na(Ⅱ)	2.280	0.04	O-Na(Ⅱ)	2.216	0.13
O-Si(Ⅱ)	1.637	0.54	O-Si(Ⅱ)	1.643	0.51	O-Si(Ⅱ)	1.691	0.50	O-Si(Ⅱ)	1.562	0.82
(**c**)											
**CuO**			**ZnO**			**PbO**			**CdO**		
**Bond**	**D(Å)**	**P**	**Bond**	**D(Å)**	**P**	**Bond**	**D(Å)**	**P**	**Bond**	**D(Å)**	**P**
Cu-O	1.879	0.29	Zn-O	1.867	0.34	Pb-O	2.198	0.18	Cd-O	2.491	0.06
Cu-Si(Ⅲ)	2.160	0.57	Zn-Si(Ⅲ)	2.338	0.65	Pb-Si(Ⅲ)	2.889	0.40	Cd-Si(Ⅲ)	2.838	0.28
O-Ca	2.237	0.12	O-Ca	2.238	0.13	O-Ca	2.242	0.13	O-Ca	2.194	0.15
O-Si(Ⅱ)	1.690	0.42	O-Si(Ⅱ)	1.766	0.37	O-Si(Ⅱ)	1.758	0.32	O-Si(Ⅱ)	1.631	0.50

Notes: D(Å)-Bond length (Å); P-Population; Cl(I) and Cl(II) represent the top and bottom Cl atoms (from chlorinated HMs) in the top view (Table A4), respectively. Na(I) and Na(II) represent the top and bottom Na atoms (from Na_2_O) in the top view (Table A4), respectively.
